# An Evaluation of Rare Cancer Policies in Europe: A Survey Among Healthcare Providers

**DOI:** 10.3390/cancers17020164

**Published:** 2025-01-07

**Authors:** Kostadin Kostadinov, Georgi Iskrov, Nina Musurlieva, Rumen Stefanov

**Affiliations:** 1 Department of Social Medicine and Public Health, Faculty of Public Health, Medical University of Plovdiv, 4002 Plovdiv, Bulgaria; georgi.iskrov@mu-plovdiv.bg (G.I.); nina.musurlieva@mu-plovdiv.bg (N.M.); rumen.stefanov@mu-plovdiv.bg (R.S.); 2 Environmental Health Division, Research Institute at Medical University of Plovdiv, 15-A “Vasil Aprilov” Blvd., 4002 Plovdiv, Bulgaria; 3 Institute for Rare Diseases, 4023 Plovdiv, Bulgaria

**Keywords:** rare cancer policies, rare cancer, national cancer plans, palliative care, health policy

## Abstract

Rare cancers, defined as those with an annual incidence of fewer than six cases per 100,000 individuals, are associated with significant health inequalities. This study aimed to assess the knowledge, attitudes, and perceptions of healthcare providers with expertise in rare cancers regarding the effectiveness of enacted or planned rare cancer policies across Europe. Between 25 March 2023 and 5 March 2024, we conducted an online survey targeting 738 healthcare providers affiliated with the European Reference Networks and the Organization of European Cancer Institutes, yielding 92 complete responses from 28 European countries (response rate: 12.5%). While a significant portion of respondents were unaware of their country’s legal definitions for rare cancers, 67.4% acknowledged that national cancer plans prioritized rare cancers. These plans received the highest ratings for their evidence-based interventions and monitoring efforts. The integration of rare cancer policies into broader oncology frameworks was the preferred policy model. National cancer registries were highly rated for confidentiality and validity but scored the lowest for cost-effectiveness. Government funding was deemed crucial for cancer screening programs. The disease burden and unmet health needs primarily influenced reimbursement decisions in the field of rare cancers. Respondents rated palliative care as more effective in adults with rare cancers compared to in children, particularly regarding symptom management. We confirmed significant variability in rare cancer policy evaluations across Europe, the necessity for a common EU-level definition for rare cancers, and a shift in reimbursement and policy framework models, highlighting the importance of policy integration and enhanced collaboration. However, given the limitations of the study, such as small sample size and possible unstudied confounding factors, we should interpret our findings with caution. A systematic policy review and multistakeholder assessment in the future could complement our results.

## Simple Summary

Rare cancers lead to significant health disparities due to difficult diagnoses and delays in accessing treatment. These cancers place a heavy burden on healthcare systems, with patients often struggling to receive timely and effective care. While many EU policies aim to support affected patients, their impact is rarely assessed, particularly from the viewpoint of healthcare providers. This study aims to assess the knowledge, attitudes, and perceived effectiveness of existing and planned rare cancer policies across Europe from the perspective of medical professionals. These findings can guide the policymaking process by enabling more targeted interventions to address patient needs effectively.

## 1. Introduction

Rare cancers are defined as those with an annual incidence of less than six per 100,000 people [1]. Although this threshold is widely accepted within the scientific community [2,3] and by some national regulatory frameworks [4], a legal definition across the EU has not yet been officially adopted [5]. According to Gatta et al. [6], rare cancers accounted for 24% of all cancer cases in the EU28 between 2000–2007. However, patients diagnosed with rare cancers often face significant health disparities, which contribute to lower 5-year survival rates compared to more common cancers [7]. Similar trends have been observed in several other countries, including the United States, Australia, Japan, and Canada, where the burden of rare cancers remains high, and survival outcomes are suboptimal [8,9,10]. These disparities are particularly evident in pediatric populations, where mortality rates for rare cancers continue to be higher compared to the more common cancers, despite significant advances in the treatment [9]. Geographic disparities in survival rates within Europe are also reported. Countries in Eastern Europe report significantly lower survival rates for rare cancers compared to their Western European counterparts, reflecting differences in healthcare and therapy access, infrastructure, and early diagnosis capabilities [6]. Comparative data on five-year net survival rates highlight additional geographical differences, showing higher overall survival estimates for all rare cancers combined in the United States compared to the European Union [11].

Like other rare diseases [12], rare cancers are marked by health discrimination [13], limited information [14,15], few research opportunities [16], inadequate preventive policies [17], diagnostic challenges [18], and fragmented care [19,20,21,22,23]. Addressing these challenges necessitates comprehensive, evidence-based strategies. Currently, two major frameworks target rare cancers. The first, Europe’s Beating Cancer Plan (EBCP) [24], prioritizes this group by investing in cancer prevention, screening, and treatment. The plan also supports research, innovation, and the improvement of therapeutic protocols, aiming to enhance access to innovative therapies. However, the EBCP does not provide specific guidelines for clinical management, recommendations for infrastructural changes, enhancement needed in the healthcare systems, nor incentives for palliative care. EBCP implementation is also limited by existing and inadequately addressed healthcare disparities among EU member states [25,26].

The second set of policies treats rare cancers as a subset of rare diseases. As a result, stakeholders of rare cancers could benefit from existing rare disease initiatives, such as the Orphan Drug Regulation [27] and the European Reference Networks (ERNs) [28,29]. The orphan drug designation offers economic incentives and accelerates regulatory approval but must also ensure equity, particularly for rare cancers where randomized clinical trials often produce limited or inconclusive results [30,31,32,33]. The scarcity of real-world post-authorization data and the limited generalizability of clinical trial outcomes exacerbate therapeutic uncertainty [34]. Despite the rise in approved innovative drugs [35], market inefficiencies for orphan drugs remain.

Market access and reimbursement criteria for rare cancer treatments differ significantly across EU member states, leading to variations in patient access to therapies [36,37,38]. These differences are influenced by national healthcare systems, economic conditions, and regulatory frameworks, affecting both the availability of treatments and the speed of their approval [39,40]. Similarly, differences exist in the structure and delivery of healthcare services, especially in managing and integrating rare cancers into broader health systems across Europe [41]. These disparities pose challenges to achieving equitable care access and highlight the importance of understanding healthcare professionals’ perceptions and the implementation of existing policies [42,43].

The aim of this study is to explore the knowledge, attitudes, and perceived effectiveness of enacted and planned rare cancer policies in Europe from the perspective of European healthcare providers.

## 2. Materials and Methods

This article adheres to the Checklist for Reporting of Survey Studies (CROSS) guidelines (Supplement File S1), to ensure a rigorous and transparent reporting of survey-based research [44].

### 2.1. Study Design

We conducted a cross-sectional online survey targeting healthcare providers working either within ERNs or the Organization of European Cancer Institutes (OECI).

#### 2.1.1. Questionnaire Development

The survey instrument (Supplement File S2), was designed exclusively for this study and was not subjected to formal validation through standard psychometric testing, as it was not intended to function as a psychometric tool. The development of the questionnaire was informed by a literature review, structured according to an epidemiological model of rare cancers. The introduction of the survey included detailed information about the study and two filter questions: one regarding informed consent and the other assessing eligibility criteria. A negative response to either of them resulted in an automatic termination of the survey form. The questionnaire consisted of 36 items, organized into five sections:

1. Responder Profile: This section aimed to capture the demographic and professional details of the respondents, such as their age, gender, years of experience in rare cancer care, type of workplace, area of specialty, and involvement in rare cancer research or treatment.
2. Overall Policies in the Field of Rare Cancers: This section sought to gather information on the existing national policies and plans related to rare cancers. It explored whether responders were aware of the legal definition of rare cancers, the presence of national cancer plans, and if these plans address rare cancers as a priority. It also examined the availability of funding sources and the extent to which stakeholders are involved in policy development. The responses were measured using a 5-point Likert scale, allowing for nuanced assessments of each aspect of the policy environment. In the scale, 5 represents strong agreement with the statement or criteria of interest and 1 indicates strong disagreement.
3. Prevention Policies: This section focused on assessing the effectiveness of current cancer prevention strategies, particularly as they relate to rare cancers. Respondents were asked to evaluate various aspects of prevention policies, including public awareness, screening programs, and the integration of rare cancers within general prevention frameworks. Key questions explored the availability of resources for prevention, the reach of awareness campaigns, and how prevention efforts are tailored for rare cancers. Like the previous section, a 5-point Likert scale was used to capture the range of responses.
4. Clinical Management, Research, and Innovation: Here, the focus was on understanding the clinical management of rare cancers, including the availability and quality of evidence-based treatment guidelines. Respondents were asked to rate various aspects of clinical collaboration, the effectiveness of clinical trials for rare cancer patients, and the funding and decision-making processes related to innovative treatment. This section also explored the level of research and innovation in rare cancer treatment, including the integration of personalized medicine and new technologies. The 5-point Likert scale was applied to assess the effectiveness and relevance of various clinical practices and strategies.
5. Palliative and Social Care: This section aimed to assess the availability and effectiveness of palliative and social support services for patients with rare cancers. Respondents were asked to evaluate the accessibility and quality of palliative care, the importance of various social support services (e.g., financial, psychosocial, and occupational), and the funding mechanisms for these services. It also considered the unique needs of pediatric and adult patients with rare cancers, focusing on the timing, quality, and patient-centeredness of the palliative care provided. A 5-point Likert scale was used to capture the importance and effectiveness of the services.

The survey instrument was pre-tested for clarity, understandability, and completion duration to ensure its relevance and alignment with the study objectives. The pre-test involved six participants: three oncologists and three researchers, selected through convenience sampling. Based on their input, no additional changes were made to the questionnaire, as it was deemed clear and suitable for the target audience. The median time to complete the survey was 7.5 min. The final version of the questionnaire was uploaded to the “LimeSurvey” platform for data collection and is publicly available at https://github.com/kostadinoff/Survey_on_rare_cancer_policies/blob/master/survey_versions/version_2.0.pdf (repository created on 21 April 2023).

The study was approved by the Ethics Committee of the Medical University of Plovdiv (approval number: 1/11 January 2024).

#### 2.1.2. Study Population

We determined the target respondent population through a two-phase process. Initially, we utilized publicly accessible lists from the websites of the European Commission [45] and OECI [46] to identify potential participants.

ERNs are collaborative networks established by the European Commission to improve the diagnosis, treatment, and care of patients with rare or complex conditions requiring specialized expertise. As of the time of the study, 24 ERNs were identified, each focusing on specific disease areas [45]. OECI is an organization aiming to promote collaboration among cancer institutes in Europe to improve cancer research, prevention, and treatment. Its membership consists of comprehensive cancer centers and research institutions from various European countries, although not all of the countries are represented equally. OECI members are recognized for their commitment to excellence in oncology and multidisciplinary care [46].

As a results of the initial search, four ERNs were deemed pertinent and selected for further analysis based on their specific focuses on rare cancers and their relevance to the study objectives: European Reference Network on Rare Adult Cancers (solid tumors)—EURACAN [47], European Reference Network on GENetic TUmour RIsk Syndromes—GENTURIS [48]; European Reference Network for Paediatric Cancer (haemato-oncology)—PaedCan [49], and European Reference Network on Rare Hematological Diseases—EuroBloodNet [50].

In the subsequent phase, we conducted an electronic search on each ERN member organization’s website to identify contact persons, including their names and email addresses. We followed the same procedure for all identified centers in the OECI database. After removing duplicate entries, we compiled and entered the contact details of 738 individuals into the respondent database within the LimeSurvey platform [51].

Eligible participants were healthcare providers affiliated with institutions that were identified as ERN, or OECI members who were actively engaged in the diagnosis, treatment, social support, or palliative care of patients with rare cancers. Exclusion criteria encompassed individuals who did not meet the eligibility requirements, explicitly refused participation, or opted to block reminder notifications regarding the survey.

We conducted the study between 25 March 2023 and 5 March 2024, and distributed it to a total of 738 participants. Each potential respondent received a personalized invitation detailing the study’s aims and objectives. We provided a unique survey link that allowed respondents to save their progress and resume later. Reminder invitations were sent on 7, 14, and 28 days, and 6 months for non-responders. We also made available a printable PDF version of the questionnaire for manual completion, and an e-mail return. In such cases, completed forms were uploaded into the LimeSurvey platform to ensure consistency in the data collection and analysis.

### 2.2. Data Quality

Out of the 738 invited participants, 144 (19.5%) accessed the online survey by opening the individual link. Among these, 3 participants (2%) formally withdrew consent and requested no further online interaction. Another 32 respondents (22.2%) provided informed consent but left all survey questions unanswered. Of the remaining 109, 8 responders (7.3%) were excluded based on ineligibility criteria, as they were not involved in the diagnosis, management, or palliative care of rare cancer patients. Incomplete survey data led to the exclusion of 9 respondents (8.9%). The final statistical analysis was performed on the fully complete responses dataset from 92 participants after this pre-analytical filtering.

### 2.3. Data Analysis

Data processing and analysis were conducted in R (version 4.4.1 [52]), with a significance level set at *p* < 0.05. Both descriptive and inferential statistical methods were employed using packages from the “Tidyverse” [53], “Tidymodels” [54], and “Easystats” [55]. Continuous variables were tested for normality with the Shapiro–Wilk test. Variables conforming to a normal distribution were described with means and standard deviations, while those not normally distributed were reported with medians and interquartile ranges. Categorical data were summarized using absolute frequencies and percentages. Differences in the distribution of respondents were assessed using the chi-square test, *t*-test, ANOVA, or the Mann–Whitney U test, as appropriate. Post-hoc tests for categorical variables were performed using the z-test with Bonferroni correction for multiple comparisons. Post-hoc tests for continuous variables were conducted using the Tukey HSD test.

The survey used a 5-point Likert scale for 18 out of 36 question items. Those responses were analyzed in two distinct ways: as continuous variables with means and standard deviations, and as ordinal variables with frequencies and percentages. The agreement with statements (agreement index) was assessed by calculating the proportion of respondents selecting “agree” (4) or “strongly agree” (5), along with half the of “neutral” (3) responses [56]. The reliability of the Likert scale items was evaluated using Cronbach’s alpha, with values exceeding 0.7 considered acceptable. Factor analysis suitability was assessed through Bartlett’s test for sphericity and the Kaiser–Meyer–Olkin (KMO) measure.

Based on the distribution of participants by country, a stratification criterion was established using the most recent data on public health expenditure per capita, adjusted for purchasing power parity (PPP) in US dollars (2022) [57]. This indicator was chosen for two primary reasons. Firstly, cancer incidence is closely related to screening practices and the resources allocated to diagnostic processes [58]. Thus, in countries with lower public health expenditure, some cancer cases may be underreported, which could obscure the relationship between health policy evaluations and actual health needs. Secondly, cancer mortality rates are significantly influenced by the effectiveness of healthcare systems [59] and levels of cardiovascular mortality [60]. The effectiveness of healthcare systems correlates with funding levels, while high cardiovascular mortality may lead to an underestimation of cancer mortality due to the potential competitive effect of earlier cardiovascular events. These assumptions emprises public health expenditure per capita as a relatively independent variable, allowing for the comparison of health policy evaluations for rare cancers in the context of fundamental health and socio-economic inequalities among participating countries [61].

## 3. Results

### 3.1. Respondent Characteristics

Responses from 92 healthcare providers across 28 countries were analyzed, resulting in a response rate of 12.5% (calculated as the number of surveys completed divided by the total number of identified responders). Italy had the highest number of participants (*n* = 10; 10.8%), followed by France (*n* = 8; 8.7%), the Czech Republic (*n* = 6; 6.5%), and the Netherlands (*n* = 6; 6.5%). Belgium, Cyprus, Finland, Ireland, Malta, Poland, and Switzerland each contributed one respondent (1.1% each). No responses were received from Albania, Ukraine, North Macedonia, Belarus, Bosnia and Herzegovina, Serbia, Montenegro, Luxembourg, or Romania (Supplement Table S1). Among the healthcare providers, 54 (58.7%) were from countries classified with higher-than-average EU public healthcare expenditure.

Table 1 provides details on demographic and professional characteristics. The median age of respondents was 51 years. Participants reported extensive overall professional experience. On average, specialized experience represented 72.5% of the total professional experience.

### 3.2. Overall Policies in the Field of Rare Cancers

#### 3.2.1. Legal Definitions and Prioritization

Most respondents (51%) indicated that their country lacked a specific legal definition for rare cancers, relying instead on the definition for rare diseases in oncology. However, 67.4% of participants indicated that rare cancers were a priority group in their NCP, with this focus being more pronounced in countries with higher public healthcare spending.

#### 3.2.2. National Cancer Plans

**Figure 1:**
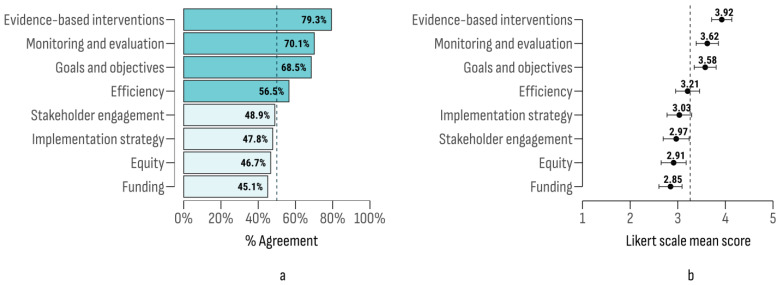
Agreement index (**a**) and Likert scale mean scores (**b**) on criteria for the analysis of NCPs.

The multi-criteria analysis evaluated the effectiveness of implemented National Cancer Plans (NCPs) across eight key dimensions: (1) the establishment of clear and measurable goals that align with overarching strategies; (2) active engagement of a diverse range of stakeholders; (3) integration of evidence-based interventions aimed at reducing cancer incidence; (4) a comprehensive and well-defined implementation strategy; (5) robust monitoring systems to track progress; (6) targeted efforts to address health disparities; (7) sufficient funding provisions; and (8) effective resource allocation. Among these, “evidence-based interventions” achieved the highest level of agreement, followed by “monitoring and evaluation” and “goals and objectives” (Figure 1).

The internal consistency of the 8 criteria as a unified construct showed good reliability (Cronbach’s α = 0.81; 95% CI [0.72, 0.87]). The KMO test indicated a high level of common factor variance (KMO = 0.82), and the Bartlett’s test for sphericity confirmed strong internal correlation (χ^2^ = 237; df = 28; *p* < 0.001). These properties facilitated the evaluation of NCPs as a single construct by aggregating Likert ratings across all criteria. The resulting aggregated score followed a normal distribution (Shapiro–Wilk test W = 0.973; *p* = 0.06) with a mean of 26.1 (95% CI [24.81, 27.38]). A significant difference in the mean multi-criteria approval was observed between countries (F (27, 64) = 2.07; *p* = 0.009). The post-hoc analysis identified the greatest agreement gap between Bulgaria and Slovenia (Δ = −17.5; 95% CI [−25.13, −9.87]; t = −4.49; *p* = 0.011) and between Bulgaria and France (Δ = −14; 95% CI [−20.61, −7.39]; t = −4.15; *p* = 0.038, Supplement Table S2)

**Figure 2:**
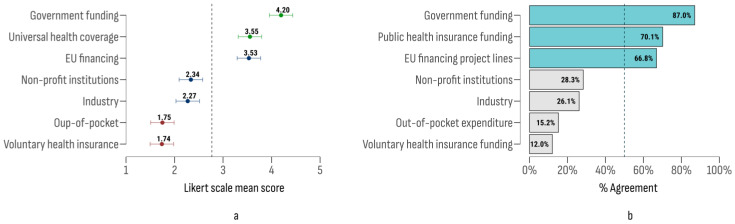
Likert scale mean scores (**a**) and agreement index (**b**) by NCP funding sources.

The assessment of funding sources for the implementation of NCPs evaluated seven distinct categories: two public (direct government expenditure and mandatory health insurance fund expenditures), two private (voluntary health insurance funds and out-of-pocket payments), and three classified as “others” (EU funds and programs, industry contributions, and NGO/crowdfunding). An ANOVA analysis (Figure 2) revealed statistically significant differences in mean ratings across funding sources (F (6637) = 63.17; *p* < 0.001). Direct government expenditure received the highest mean rating, followed by mandatory health insurance fund expenditures and EU funds and programs. Significant differences were observed between direct government expenditure and voluntary health insurance funds (Δ = 2.46; 95% CI [2.13, 2.79]; t = 14.08; *p* < 0.001), as well as between direct government expenditure and out-of-pocket payments (Δ = 2.45; 95% CI [2.12, 2.78]; t = 14.02; *p* < 0.001).

#### 3.2.3. Rare Cancer Policies Integration

The final item in this section evaluated how rare cancer policies should be aligned with broader health policies. Respondents chose between three options: integrating rare cancer policies into general oncology policies, aligning them with rare disease policies, or establishing a separate policy area. The majority preferred integrating rare cancer policies into general oncology policies (*n* = 46; 50%, 95% CI [39.4%, 60.6%]).

### 3.3. Effectiveness of National Cancer Registries and Prevention Policies

#### 3.3.1. National Cancer Registries

This survey section assessed NCR effectiveness across twelve criteria: (1) completeness—recording all cancer cases within a defined population and time period; (2) contemporaneity—ensuring data is timely and current; (3) accuracy—ensuring data reliability; (4) validity—providing dependable statistics on cancer incidence, mortality, and survival; (5) representativeness—accurately reflecting the target population; (6) confidentiality—protecting individual privacy; (7) influence on patient outcomes; (8) informing cancer policy decisions; (9) contribution to cancer research; (10) cost-effectiveness; (11) impact on public awareness; and (12) fostering collaboration among healthcare providers, researchers, and public health officials.

**Figure 3:**
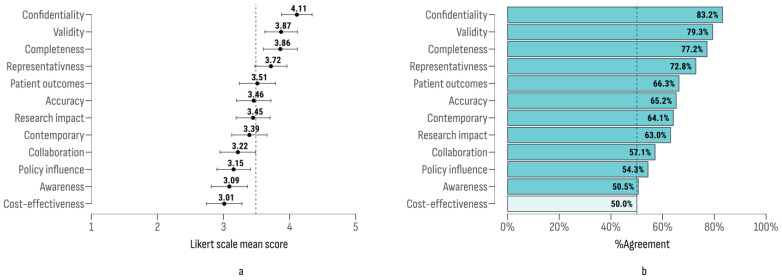
Likert scale mean scores (**a**) and agreement index (**b**) on NCR assessment criteria.

The results (Figure 3a) indicated that “confidentiality” received the highest agreement score. The largest gap in agreement was between “confidentiality” and “cost-effectiveness” (Δ = 1.1; 95% CI [0.79, 1.41]; t = 5.89; *p* < 0.001), with “cost-effectiveness” being the only criterion rated at a neutral agreement level (Figure 3b).

The criteria exhibited excellent internal consistency and reliability (Cronbach’s α = 0.918; 95% CI [0.90, 0.94]). The KMO test indicated a high measure of sampling adequacy (MSA = 0.9), and BTS (χ^2^ = 641; df = 66; *p* < 0.01) confirmed the appropriateness of aggregating ratings across all criteria. The resulting composite variable, reflecting the multi-criteria approval of cancer registries, had a range from 12 to 60, with a mean score of 41.8 (± 11).

A significant relationship was observed between the respondent’s country and their multi-criteria score for cancer registry effectiveness (F (27, 64) = 6.28; *p* < 0.001). Belgium (x = 58), Slovenia (x = 57), and the Czech Republic (x = 51.8) received the highest ratings, while the Bulgarian cancer registry scored the lowest (x = 14).

#### 3.3.2. Screening Policies

**Figure 4:**
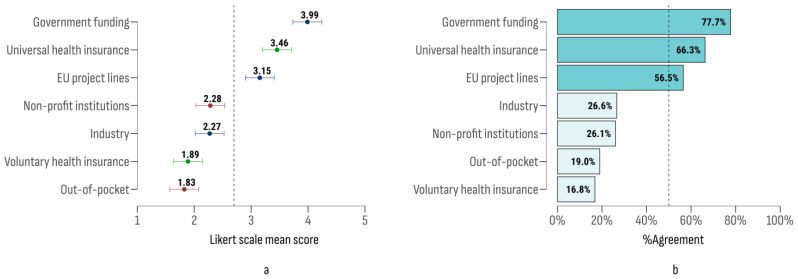
**The** Likert scale mean scores (**a**) and agreement index (**b**) on funding sources for screening programs.

The study section also evaluated expert opinions on the funding for screening programs, maintaining the same sources as those assessed in the NCP section. The results are depicted in Figure 4. Government funding was rated with the highest importance, followed by health insurance funds and EU project funds.

Significant differences in agreement were identified across the sources studied (*F* (6, 610) = 44.47; *p* < 0.001). The most prominent post-hoc differences were between government funding and out-of-pocket payments (Δ = 2.16; 95% CI [1.81, 2.51]; t = 11.84; *p* < 0.001) and between government and industry funding (Δ = 1.72; 95% CI [1.37, 2.07]; t = 9.40; *p* < 0.001).

### 3.4. Clinical Management

#### 3.4.1. Diagnostic-Therapeutic Guidelines

**Figure 5:**
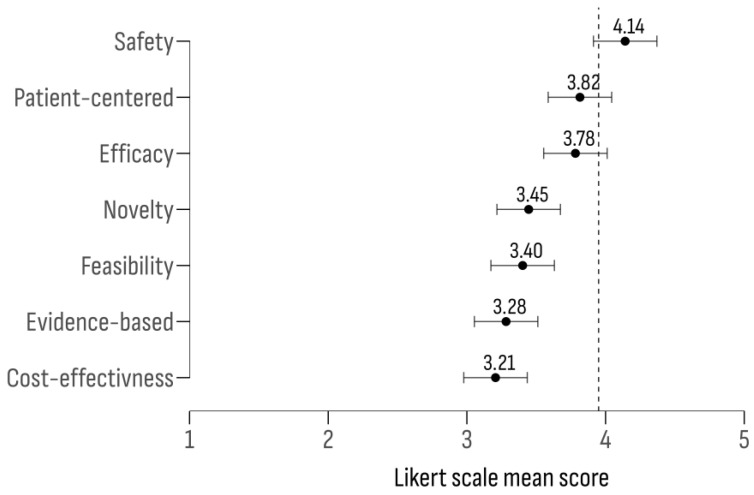
Likert scale mean scores for DTGs efficacy criteria.

To evaluate the effectiveness of Diagnostic and Therapeutic Guidelines (DTGs), seven key criteria were analyzed as follows: (1) safety—ensuring minimal and predictable side effects; (2) clinical effectiveness—demonstrated efficacy in treating rare cancers; (3) evidence base—backed by strong scientific data; (4) patient-centeredness—enabling tailored decision-making for individual patients; (5) applicability—ease of implementation in clinical settings; (6) cost-effectiveness—balancing costs with benefits; and (7) novelty—incorporating innovative treatment methods. The results (Figure 5) highlighted “safety” as the most important criterion, followed by “patient-centeredness” and “clinical effectiveness”.

A moderate and significant relationship was observed between the evaluated criteria and their assigned ratings (F (6, 637) = 8.42; *p* < 0.01). The post-hoc analyses revealed significant discrepancies between “safety” and “cost-effectiveness” (Δ = 0.93; 95% CI [0.60, 1.26]; *p* < 0.01) and between “safety” and “evidence base” (Δ = 0.86; 95% CI [0.53, 1.19]; *p* < 0.01).

**Figure 6:**
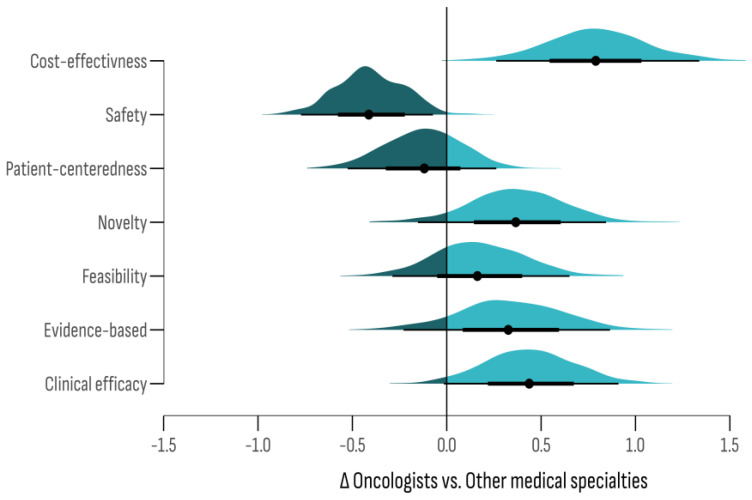
The difference in ratings by criteria for DTG effectiveness—a comparison between “oncologists” and “other” specialists.

Differences in ratings by criteria were further analyzed based on respondents’ specialty, comparing “oncologists” with “other” specialists. A bootstrap resampling simulation with 1000 iterations (Figure 6) indicated that in 98.1% of the iterations, “safety” received a lower importance rating among oncologists compared to other specialists. Additionally, a high probability of a negative difference was observed for “patient-centeredness” (P_Δ<0_ = 73.6%). Conversely, “cost-effectiveness” (P_Δ>0_ = 100%), “clinical effectiveness” (P_Δ>0_ = 97.2%), and “novelty” (P_Δ>0_ = 92.6%) had high probabilities of being rated with a greater importance by oncologists.

#### 3.4.2. Rare Cancer Treatment

**Figure 7:**
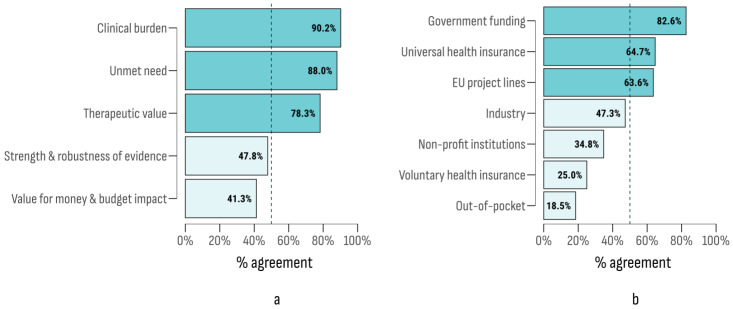
Agreement index of factors affecting the public reimbursement decisions (**a**) and funding sources (**b**).

Rare cancer treatment was analyzed from two perspectives. Firstly, we assessed the importance of six factors influencing reimbursement decisions: (1) disease burden, (2) unmet health needs (defined as a lack of therapeutic alternatives), (3) quality of scientific data, (4) costs and budget impact, and (5) therapeutic value. The second perspective examined the significance of the seven funding sources previously discussed. The results (Figure 7) identified three factors with agreement indices above 50%. The most important criterion was the disease burden. Among the funding sources, those with approval rates exceeding the agreement threshold were direct government spending, mandatory universal health insurance, and EU programs and funds.

**Figure 8:**
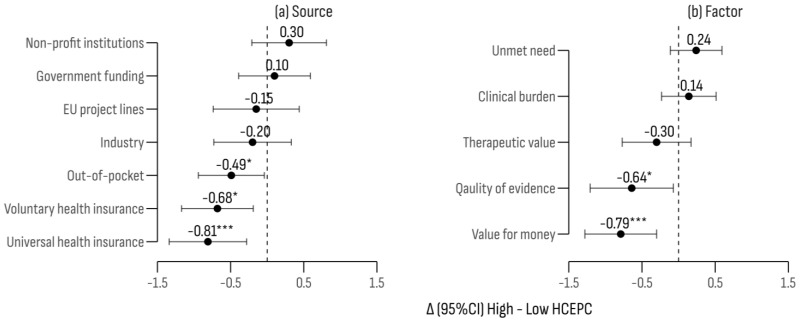
A comparison of differences in ratings for funding sources and factors by healthcare expenditure per capita. Significance levels: * *p* < 0.05; *** *p* < 0.001.

Evaluations of reimbursement factors and funding sources were influenced by the country’s level of healthcare expenditure (Figure 8; F (4, 428) = 51.26; *p* < 0.001). The most significant discrepancies were observed for “Universal Health Insurance” (Δ = −0.81; 95% CI [−1.34, −0.28]; *p* < 0.01) and “budgetary impact” (Δ = −0.79; 95% CI [−1.28, −0.30]; *p* < 0.01), with both rated higher by respondents from countries classified with a lower-then-average healthcare expenditure.

### 3.5. Palliative Care

**Figure 9:**
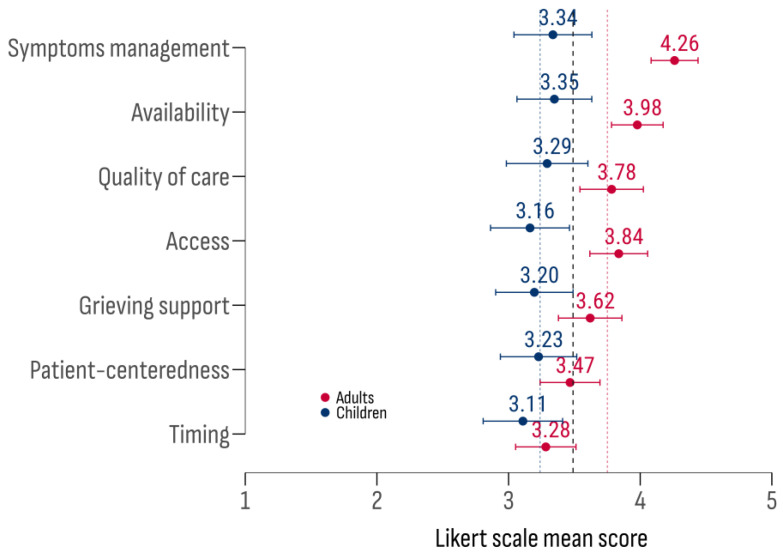
Likert scale mean scores ratings for the effectiveness of palliative care.

The effectiveness of palliative care was assessed using seven criteria: (1) availability—presence of palliative care services for patients with rare cancers; (2) accessibility—ease of access regardless of residence or socio-economic status; (3) quality—comprehensive care by a multidisciplinary team based on proven interventions; (4) timeliness—alignment of care initiation with illness progression; (5) personalization—delivery of care according to a personalized plan, including shared decision-making and consideration of social and cultural factors; (6) symptom management—effective management of pain, nausea, vomiting, fatigue, and other symptoms; and (7) bereavement support—support for families after the patient’s death, including psychological counseling and support groups. Respondents evaluated the criteria set separately for adults and children (Figure 9).

**Figure 10:**
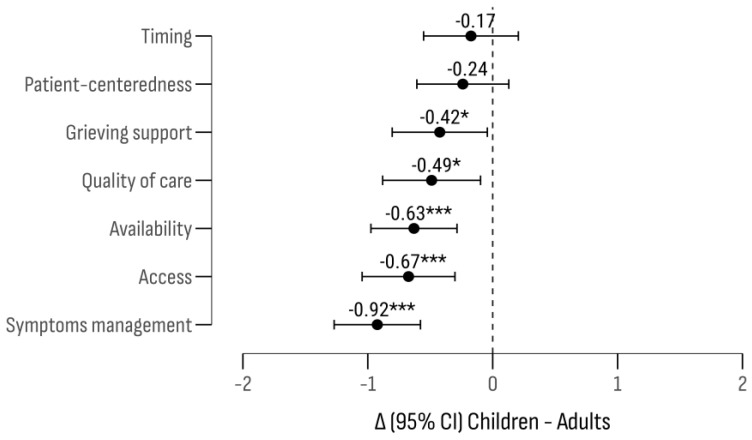
The difference in criterion scores by age group of rare cancer patients (children–adults). The X axis represents the Likert score rating difference between children and adults. The Y axis represents the criteria set. Significance levels: * *p* < 0.05; *** *p* < 0.001.

The highest approval rating was given to “symptom management”, followed by “availability” and “accessibility”. The lowest ratings were for “timeliness” and “personalization”. Significant differences were noted between patient groups (F (1, 1286) = 49.82; *p* < 0.001), with higher effectiveness ratings for adults in “symptom management”, “accessibility”, “availability”, “quality”, and “bereavement support” (Figure 10).

**Figure 11:**
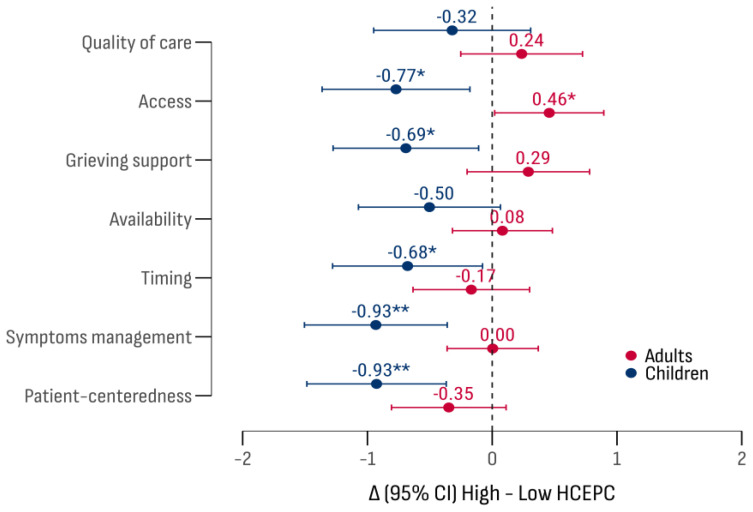
A comparison of evaluation differences by the criteria and age groups relative to public healthcare expenditure per capita. Significance levels: * *p* < 0.05; ** *p* < 0.01.

An analysis of factors influencing the estimates revealed that public health expenditure per capita was a significant stratification criterion (F (1, 1278) = 18.36; *p* < 0.001), with a notable interaction with patient age group (F (1, 1278) = 29.10; *p* < 0.001;). The results indicated that experts in countries with higher healthcare expenditure rated the “accessibility” of palliative care for children more positively than adults. On the other hand, experts from countries with lower healthcare spending rated the “accessibility” higher for adults. Lower scores for adults were noted for “timeliness”, “bereavement support”, “personalization”, and “symptom management” (Figure 11).

The analysis of funding alternatives for palliative care identified only two sources with agreement levels exceeding a 50% threshold—government spending and universal health insurance funds.

## 4. Discussion

We confirmed significant variability in rare cancer policy evaluations across Europe, the necessity for a common EU-level definition for rare cancers, and a shift in reimbursement and policy framework models, highlighting the importance of policy integration and enhanced collaboration. However, given the limitations of the study, we should interpret our findings with caution. We intentionally designed our study to measure the attitudes, knowledge, and perceptions of rare cancer medical experts in Europe. This is, of course, a subjective personal evaluation, which may be influenced by unstudied confounding factors such as personal biases. On the other hand, a policy document review may describe rare cancer policies in more detail. However, the subsequent policy outcomes could often substantially deviate from the original policy goals [62,63]. This is why we explicitly decided to focus on the medical experts’ perspective as a reliable source of rare cancer policy experience and expertise.

### 4.1. Variability in Defining Rare Tumors

The definition of rare cancer is a cornerstone in developing policies, as it delineates the scope, identifies population health needs, and informs resource allocation [64,65]. Unlike rare diseases, which are defined by a prevalence threshold (<5/10,000), the definition of rare cancers is based on incidence rates (<6/100,000), reflecting the unique epidemiological characteristics of these conditions, including their disproportionate burden during the first year after diagnosis [64]. The international scientific community has endorsed this incidence-based definition as a standard for rare cancer research and policy formation [66].

Our findings reveal considerable variability in the respondents’ awareness of a legal definition of rare cancer within the national healthcare policy framework. Over half of the participants reported using the definition for rare diseases in oncology rather than the specific rare cancer definition. However, respondents’ awareness may not fully align with the actual legal framework, which could lead to three possible discussion scenarios. Firstly, respondents’ awareness might reflect the actual legal framework, a plausible assumption given the participants’ expertise in rare cancer care. Alternatively, responders might report “false” awareness, believing the specific definition exists when it in fact does not. This is particularly relevant in countries where new cancer strategies adopt the definition for rare cancer without any corresponding legislative change. For example, in Bulgaria, the National Cancer Control Program (2023–2027) incorporates the proposed definition based on incidence rates (<6/100,000), but the legal framework continues to rely on the prevalence threshold for rare diseases in oncology (<5/10,000) [67,68]. Lastly, respondents may be unaware of an existing specific legal definition due to the limited impact on clinical practice, such as when the implementation of the definition does not influence reimbursement policies, treatment guidelines, or research practices.

Regardless of the scenario, addressing the variability in definitions within the field is recognized as a significant challenge at the EU level. Consequently, many policy re-searchers and stakeholders advocate for a harmonized definition that differentiates rare cancers from rare diseases, facilitating the development of tailored health policies and preventing the suppression of rare cancer-specific needs within broader rare disease strategies [69].

### 4.2. Critical Elements and Challenges in National Cancer Plans

Once a definition is established, the policy framework for rare cancers is usually integrated into a part of the national cancer plans (NCPs). Our survey identified several perceived critical elements for effective cancer plans in the field of rare cancer, including evidence-based interventions, well-defined goals and objectives, measurable performance indicators, and robust monitoring and evaluation mechanisms. However, based on the responders’ evaluation, we also observed challenges such as inadequate funding, ambiguous implementation strategies, and widening health inequalities. It should be noted that the evaluation of NCPs is based on subjective expert opinions, which reflects only the perceived effectiveness of these policies in real-world settings. More objective evaluation would require a comprehensive analysis of the actual documentation, their stage of implementation, and measurable epidemiological and clinical outcomes. Nevertheless, the findings underscore the importance of aligning NCPs with the specific needs of rare cancer patients and reflect other studies indicating that EU oncology policies often fall short in addressing innovation and providing financial solutions for new oncological therapies [70,71].

A significant difference was also observed in the evaluation of the effectiveness of national cancer plans relative to county-level public health expenditure per capita. The higher overall rating among respondents from countries with higher public health spending may be attributed to two major factors. Firstly, these countries often involve patient organizations more extensively in the development of cancer plans [72], ensuring that measures are more closely aligned with the practical needs of patients and their families. Secondly, economically developed countries are more likely to include comprehensive cancer policies addressing social determinants of health and regional disparities, thereby enhancing the specificity and responsiveness of their NCPs [71,73].

Experts from countries with a higher-than-average public health expenditure rated financial sources other than government funding as significantly higher. This trend reflects the reliance on alternative funding mechanisms, such as out-of-pocket expenses and voluntary health insurance schemes, to implement cancer control measures. A recent study [74] provides a possible explanation for this trend, suggesting that in the high public health spending countries, patient co-payments are viewed as supplementary funding sources, engaging in personal responsibility. In contrast, in countries with low public health spending, patient co-payments are primarily seen as a way to compensate for dissatisfaction with underfunded public health services. As a result, in these countries, the importance of government spending is emphasized, particularly in the implementation of strategic healthcare plans.

### 4.3. National Cancer Registries and Screening Programs

National cancer registries (NCRs) are essential for monitoring cancer incidence, prevalence, and survival rates, as well as for evaluating the effectiveness of cancer control programs [75]. Our study identified several key criteria for assessing NCRs in the context of rare cancer. Among the experts, “confidentiality” and “validity” received the highest approval ratings, aligning with findings from previous evaluations of cancer registry quality [76,77]. Notably, the criterion of “cost-effectiveness” was rated as neutral. These results underscore the importance of adhering to high-quality standards for cancer registries, including regular updates and comprehensive data collection, despite the lack of direct evidence linking these factors to public policy outcomes [78]. In this context, government funding emerged as a crucial element for ensuring the sustainability, reliability, and validity of NCRs [79,80].

In our study, three funding sources for screening programs received approval rates above the midpoint threshold: government funding, health insurance funds, and EU programs and funds. These endorsements highlight an awareness of the primary financial challenges faced in implementing screening programs within the EU. Researchers identified patient co-payments as a significant barrier to access and an exacerbation of health inequalities [61,81]. Even with comprehensive funding from health insurance, barriers such as religious and cultural beliefs, high levels of anxiety, and logistical issues persist [82]. These findings suggest that government funding is crucial for ensuring the sustainability and expansion of organized screening initiatives. Additionally, recent evidence indicates that government funding is associated with increased screening participation and improved health outcomes [83]. However, reliance solely on this source may not be sufficient to address emerging health needs, particularly with advancements like whole genome sequencing [84,85]. Experts working with children support this by rating private funding sources, such as voluntary health insurance and direct patient co-payments, significantly higher.

### 4.4. Clinical Management and Innovative Treatments

The assessment of diagnostic and therapeutic guidelines revealed the highest approval ratings for “safety”, “patient-centeredness”, and “clinical effectiveness”. However, the sub-group analysis indicated that oncologists assigned a higher score to “novelty”, “cost-effectiveness”, and “evidence base”. Oncologists’ focus on achieving significant therapeutic goals, such as complete cures or extended survival rates, may account for this preference, potentially at the expense of increased adverse effects [86]. The tension seen between “patient-centeredness” and “evidence-based interventions” is in line with what other studies have found, which is that patient-centered care approaches often go against established quality and safety standards [87,88]. This discrepancy underscores the challenge of reconciling individual patient preferences with the rigorous demands of evidence-based practice.

The findings on reimbursement factors for innovative oncology treatments underscore a significant issue facing researchers and health authorities. Delays in accessing new therapies are common, even in advanced economies [40,89]. Moreover, reimbursed therapies often lack robust evidence of significant improvements in survival or quality of life [90]. This fuels the debate on the most effective financial mechanisms for reimbursement, aiming to balance rapid access with a careful management of public resources [91,92,93]. In our study, the most highly agreed-upon factor for reimbursement was disease severity, followed by unmet clinical needs. Among funding sources, “direct government spending” and “mandatory health insurance funds” received the highest approval. Notably, respondents from countries with lower public healthcare expenditures rated “budget impact” and “quality of scientific data” more highly, aligning with research emphasizing the use of stricter reimbursement criteria in countries with lower public spending [39,94,95].

### 4.5. Palliative Care

Our results identified a significant disparity in the effectiveness of palliative care provided to children, particularly for criteria such as “symptom management”, “availability”, and “accessibility.” This issue has been recognized in other research, which highlights factors such as late referrals to specialized palliative care teams, resistance from oncologists and hematologists to initiate palliative therapy, psychological and social barriers, and organizational obstacles within health systems [43,96,97,98,99]. The criterion “timeliness” was rated the lowest for both adults and children. These findings emphasize the importance of early referrals and the integration of palliative care, even during ongoing active oncology treatment [100]. Furthermore, expert evaluations of effectiveness showed significant territorial differences based on the respondent’s country and the level of public healthcare spending. This highlights the need for the establishment of common European quality and integration standards, as well as guidelines for effective financial support for these activities [101,102].

An interesting pattern emerged regarding palliative care funding. Without public funding, patients often experience longer hospital stays and higher costs, including those from informal caregiving [103,104]. On the other hand, when palliative care is funded for home or hospice settings, it leads to lower hospital costs and less strain on family members [43,105]. The survey results reflect these trends, showing the strongest support for “government funding” and mandatory health insurance funds, leaving other sources without any significant approval.

## 5. Limitations

There are several important limitations to consider when interpreting the results of our study. Firstly, the survey sample was relatively small, which may limit its representativeness of the broader population of medical experts in rare cancers across the EU. However, the respondents’ diverse demographic and professional characteristics, including various ages, years of experience, specializations, countries of residence, and academic roles may help offset some of the limitations related to the sample size. Furthermore, while the low response rate in our study may limit generalizability, the targeted nature of the sample, which focused on healthcare providers directly involved in rare cancer care, provides valuable insights into the perspectives of this specialized group. Although future studies could benefit from larger sample sizes, the data collected in this study still offers meaningful perspectives on the challenges and opportunities in rare cancer care across Europe.

Secondly, the survey was conducted online in English, which may have introduced selection bias. This language constraint could have excluded potential respondents who were not proficient in English, potentially affecting the diversity of the viewpoints represented. Additionally, the online format might have limited the participation from individuals with limited access to digital platforms or those who prefer face-to-face interactions.

Thirdly, the survey used a cross-sectional design, which limits the ability to draw causal inferences from the results. Our research captures opinions and evaluations at a single point in time, and thus does not reflect changes in attitudes or perspectives over time or when concrete new policies are implemented. In future studies, a longitudinal design based on the presented methodology could provide more robust evidence.

Fourthly, it is important to note that the survey primarily measures respondents’ subjective opinions, which are shaped by their professional experience, knowledge, and beliefs. These opinions may not always align with objective policy outcomes or other measures of effectiveness. Moreover, they could be influenced by unstudied confounding factors such as personal biases, and professional or political views. Future research should address this limitation by incorporating a systematic analysis of policy documents alongside stakeholder evaluations to provide a more comprehensive and objective assessment of their alignment and impact.

Fifthly, although a pilot test was administered, the questionnaire used in this study has not been fully validated. Specifically, test-retest reliability and construct validity were not assessed through comparisons to other established scales, as no instruments measuring policy implementation in the field of rare cancers, similar to those used in this questionnaire, were identified.

Lastly, disparities in expertise among specialists across the diverse fields covered in this questionnaire may have also influenced their evaluations of the policies and practices discussed. Future research should aim to include the perspectives of a broader range of stakeholders, such as patients, health authorities, and payers, to gain a more comprehensive understanding of these issues. Such an inclusive approach would provide valuable insights into the multifaceted challenges and opportunities in developing effective oncology policies.

## 6. Conclusions

This study provides valuable insights into the evaluation of rare cancer policies in the EU as assessed by experts in the field. It underscores the impact of demographic and professional factors on the evaluation of NCPs, highlights the critical role of funding sources in policy implementation, and emphasizes the importance of national cancer registries. Additionally, it examines the effectiveness of screening programs and diagnostic and therapeutic guidelines, explores factors influencing reimbursement decisions, and evaluates palliative care effectiveness. Significant disparities were observed in policy evaluations based on public healthcare expenditure per capita and patient age groups. These findings offer a foundation for further research and the development of more effective policies for managing rare cancers in the EU.

## Figures and Tables

**Table 1:** The demographic and professional characteristics of study participants.

Characteristic	*n* = 92
Age (in years, Median, and IQR)	51 (47, 60)
Sex (male)	41 (45%)
Experience in the field of rare cancers (in years, Median, and IQR)	18 (12, 25)
Overall professional experience (in years, Median, and IQR)	24 (20, 32)
Workplace	
Healthcare institutions	70 (76%)
Health authorities	2 (2.2%)
University	43 (47%)
Research organization	29 (32%)
Other	2 (2.2%)
Specialization	
Oncology	55 (60%)
Hematology	14 (15%)
Surgery	11 (12%)
Pediatric oncologic hematology	12 (13%)
Non-medical specialty	4 (4.3%)
Radiotherapy	7 (7.6%)
Medical genetics	3 (3.3%)
Pathology	3 (3.3%)
Other specialty	2 (2.2%)
Endocrinology	3 (3.3%)
Internal medicine	3 (3.3%)
Public health	1 (1.1%)
Patient Groups Managed	
Adults	63 (68%)
Children	22 (24%)
Adults and children	7 (7.6%)
Primary Area of Expertise	
Sarcomas	34 (37%)
Rare cancers in children	23 (25%)
Gastrointestinal tract	23 (25%)
Neuroendocrine cancers	18 (20%)
Central nervous system	16 (17%)
Endocrine organs	16 (17%)
Female reproductive organs	15 (16%)
Rare thoracic cancers	15 (16%)
Hematological types	14 (15%)
Head and neck	13 (14%)
Rare skin cancers	11 (12%)
Male reproductive system and urogenital rare cancers	7 (7.6%)
European Reference Network	
EUROCAN	41 (45%)
PaedCan	23 (25%)
EuroBloodNet	14 (15%)
GENTURIS	15 (16%)
No affiliation with ERN	10 (11%)
Primary Activity	
Clinical diagnosis and treatment	78 (85%)
Research	6 (6.5%)
Laboratory, genetic, and pathological diagnostics	4 (4.3%)
Prevention	3 (3.3%)
Social support, rehabilitation, and palliative care	1 (1.1%)

## Data Availability

The survey instrument and the data can be found at https://github.com/kostadinoff/Survey_on_rare_cancer_policies (created on 21 April 2023).

## Supplementary Materials

The following supporting information can be downloaded at: https://www.mdpi.com/article/10.3390/cancers17020164/s1, Table S1: Number of respondents per country; Table S2: Multi-criteria scoring of cancer plan effectiveness across countries; File S1: Checklist for Reporting Of Survey Studies (CROSS); File S2: The survey instrument.
