# Supplementary material for: An Evaluation of Rare Cancer Policies in Europe: A Survey Among Healthcare Providers
Source: Cancers (Basel). 2025 Jan 7;17(2):164. doi: 10.3390/cancers17020164 (PMC11764363; doi:10.3390/cancers17020164)
Supplement: Supplementary file 1 [file cancers-17-00164-s001.zip › S2.pdf]

LimeSurvey

Section A: INFORMED CONSENT

A1. **I have had enough time to consider my participation in the study.**

I have received sufficient information about my rights and purpose of the study and its implementation, as well as about the possible benefits and disadvantages related to it.

I have not been pressured or persuaded to participate in the study.

I know that my information will be treated confidentially.

I understand that my participation is voluntary.

By clicking Yes, I confirm my participation in this study and agree to be a voluntary research subject.

Yes ☐

No ☐

A2. **Are you responsible for the diagnosis, prevention, research, or clinical management of patients with rare cancers?**

Yes ☐

No ☐

Section B: RESPONDER PROFILE

B1. **Please answer the following questions about yourself:**

Age (years) ☐☐☐☐☐☐☐☐☐☐

Country ☐☐☐☐☐☐☐☐☐☐

What is the duration of your personal involvement in the field rare cancers? /in years/ ☐☐☐☐☐☐☐☐☐☐

What is the duration of your overall working experience? /in years/ ☐☐☐☐☐☐☐☐☐☐

B2. **What is your gender?**

Female ☐

Male ☐

B3. **Workplace type:**

Medical establishment ☐

Health authority ☐

University ☐

Research organisation ☐

Other ☐

Other

B4. What is your specialty?

Oncology ☐

Hematology ☐

Surgery ☐

Radiotherapy ☐

Nuclear medicine ☐

(Medical) genetic ☐

Histopathologists ☐

Not a medical doctor ☐

Other ☐

Other

B5. Which age group of patients with rare tumors is the focus of your work?

Children ☐

Adults ☐

Both ☐

B6. In what area(s) of rare cancers are you specialized?

Central nervous system rare cancers (CNS) ☐

Digestive rare cancers ☐

Endocrine organ rare cancers ☐

Female genital rare cancers ☐

Haematological rare cancers ☐

Head & neck rare cancers ☐

Male genital & urogenital rare cancers ☐

Neuroendocrine tumours (net) rare cancers ☐

Paediatric cancers ☐

Sarcomas ☐

Skin rare cancers ☐

Thoracic rare cancers ☐

Other ☐

Other

B7. **In which of the following <u>ERNs</u> are you and your organization involved?**

EURACAN ☐

ERN PaedCan ☐

ERN-EuroBloodNet ☐

ERN GENTURIS ☐

None ☐

Other ☐

Other

B8. What is the <u>main focus</u> of your professional activities in the field of rare cancers?

- Laboratory, genetic or histopathological diagnosis ☐
- Clinical diagnosis and management ☐
- Research and innovation ☐
- Prevention ☐
- Social support, rehabilitation and palliative care ☐
- Other (please specify in the comment box) ☐

Section C: OVERALL POLICIES IN THE FIELD OF RARE CANCERS

C1. Is there a <u>legal definition</u> of rare cancers in your country?

- Yes ☐
- No, but the general definition for rare diseases is applied. ☐
- No ☐

C2. Is there <u>currently</u> an adopted <u>national cancer plan</u> in place in your country?

- Yes ☐
- No ☐

C3. Are rare cancers defined as <u>a priority group</u> in the national cancer plan?

- Yes ☐
- No ☐

LimeSurvey

C4. How would you assess the <u>role and relative impact of the National Cancer Plan</u> based on the following criteria in your country?

Please assess each criterion on a scale of 1 to 5, with 1 indicating strongly disagree and 5 indicating strongly agree.

	1	2	3	4	5
GOALS AND OBJECTIVES: The plan has a clear, achievable and measurable goals and objectives that are aligned with the overall strategy.	☐	☐	☐	☐	☐
STAKEHOLDER ENGAGEMNT: The plan involves a broad range of stakeholders, including patients, healthcare providers, researchers, policymakers, and advocacy groups.	☐	☐	☐	☐	☐
EVIDENCE-BASED INTERVENTIONS: The plan includes interventions that have been shown to be effective in reducing the burden of cancer.	☐	☐	☐	☐	☐
IMPLEMENTATION STRATEGY: The plan has a clear implementation strategy that outlines how the goals and objectives will be achieved.	☐	☐	☐	☐	☐
MONITORING AND EVALUATION: The plan includes a framework to track progress towards the goals and objectives.	☐	☐	☐	☐	☐
EQUITY: The plan addresses health disparities and includes strategies to reduce barriers to care, such as geographic or financial barriers.	☐	☐	☐	☐	☐
FUNDING: The plan is adequately funded to ensure that the goals and objectives are achieved.	☐	☐	☐	☐	☐
EFFICIENCY: The financing of the plan is efficient, ensuring that resources are used effectively to achieve the goals and objectives of the plan.	☐	☐	☐	☐	☐

C5. How would you assess the importance of different <u>funding sources for the National Cancer Plan</u> in your country?

This question is not asking for your subjective opinion but rather about the active cancer plan. Please rate each source on a scale of 1 to 5, with 1 indicating that the resource is not contributing to the plan and 5 indicating that the resource is a primary source of funding.

	1	2	3	4	5
Public - Goverment funding	☐	☐	☐	☐	☐
Public - Health insurance funding	☐	☐	☐	☐	☐
Private - Voluntary health insurance funding	☐	☐	☐	☐	☐
Private - Household out-of- pocket expenditure	☐	☐	☐	☐	☐
Other - Non-profit institutions serving households	☐	☐	☐	☐	☐
Other - EU financing project lines	☐	☐	☐	☐	☐
Other - Industry	☐	☐	☐	☐	☐

C6. Which of the following approaches for rare cancer policies integration do you find most suitable?

- [ ] Aligning rare cancer policies with all other cancer-related policies
- [ ] Aligning rare cancer policies with other policies related to rare diseases
- [ ] Establishing rare cancer policies as a distinct area of policies

Section D: PREVENTION POLICIES

D1. How would you assess the effectiveness of the established cancer prevention policies (including those for rare cancers) in your country based on the following criteria?

Please use the scale from 1 to 5, where 1 indicates strongly disagree and 5 indicates strongly agree

	1	2	3	4	5
KNOWLEDGE: There is a basic understanding of what rare cancers are, how they differ from common cancers, and their impact on individuals and society.	☐	☐	☐	☐	☐
PERCEPTION: Rare cancers are perceived as a significant health issue. The unique challenges faced by patients with rare cancers are understood. The need for increased research, funding, and policy support is recognized.	☐	☐	☐	☐	☐
ATTITUDE: The attitude towards patients with rare cancers is supportive. The society is open to learning more about rare cancers and advocating for increased awareness and support.	☐	☐	☐	☐	☐
COMMUNICATION: Effective communication strategies are in place to educate society about rare cancers.	☐	☐	☐	☐	☐
REACH: Awareness campaigns reach a broad range of society, including different age groups, genders, socioeconomic backgrounds, and geographic locations.	☐	☐	☐	☐	☐
IMPACT: The impact of prevention campaigns is measured through indicators such as changes in incidence and mortality from rare cancers. The campaigns have an impact on the allocation of resources, policy development, and healthcare delivery for patients with rare cancers.	☐	☐	☐	☐	☐

LimeSurvey

D2. How would you assess the <u>effectiveness of the national cancer registry</u> in your country based on the following criteria?

Please use the scale from 1 to 5, where 1 indicates strongly disagree and 5 indicates strongly agree. If there is no national but only a regional registry in place, answer accordingly.

	1	2	3	4	5
COMPLETENESS: The registry includes all cases of cancer diagnosed within a defined population and time period.	☐	☐	☐	☐	☐
CONTEMPORARY: The registry has up-to-date and timely data on cancer cases.	☐	☐	☐	☐	☐
ACCURACY: The data collected in the registry is accurate and reliable.	☐	☐	☐	☐	☐
VALIDITY: The registry is able to produce valid statistics on cancer incidence, mortality, and survival.	☐	☐	☐	☐	☐
REPRESENTATIVNESS: The registry is representative of the population it serves.	☐	☐	☐	☐	☐
CONFIDENTIALITY: The registry ensures the confidentiality and privacy of the individuals whose data are collected	☐	☐	☐	☐	☐
PATIENT OUTCOMES: The registry demonstrates a positive impact on patient outcomes, such as earlier diagnosis, more effective treatment, and improved survival rates.	☐	☐	☐	☐	☐
INFLUENCE: The registry informs cancer policy decisions.	☐	☐	☐	☐	☐
RESEARCH: The registry facilitates cancer research by providing data and resources for investigators	☐	☐	☐	☐	☐
COST-EFFECTIVNESS: The registry demonstrates that its benefits outweigh its costs.	☐	☐	☐	☐	☐
AWARENESS: The registry increases public awareness of cancer and its impact on the population.	☐	☐	☐	☐	☐
COLLABORATION: The registry facilitates collaboration among healthcare providers, researchers, and public health officials.	☐	☐	☐	☐	☐

D3. How would you assess the <u>importance of different funding sources</u> for screening programs in your country for patients with rare cancers?

This question is <u>not seeking your subjective opinion but is about the current screening programs</u>. Please rate each source on a scale of 1 to 5, with 1 indicating that the resource is not used and 5 indicating that the resource is a primary source of funding.

	1	2	3	4	5
Public - Goverment funding	☐	☐	☐	☐	☐
Public - Health insurance funding	☐	☐	☐	☐	☐
Private - Voluntary health insurance funding	☐	☐	☐	☐	☐
Private - Household out-of- pocket expenditure	☐	☐	☐	☐	☐
Other - Non-profit institutions serving households	☐	☐	☐	☐	☐
Other - EU financing project lines	☐	☐	☐	☐	☐
Other - Industry	☐	☐	☐	☐	☐

LimeSurvey

D4. How would you assess the implementation of the following screening programs established by the EU Council recommendation on strengthening prevention through early detection in your country?

The implementation of a screening program refers to the process of putting into action a planned and organized set of activities aimed at identifying a specific disease or condition in a target population through the use of a specific screening intervention. Please use the scale from 1 to 5, with 1 denoting no implementation and 5 denoting complete implementation.

	1	2	3	4	5
Breast cancer screening with mammography for women aged 50 to 69, and suggested for women from 45 to 74 years old	☐	☐	☐	☐	☐
Testing for human papilloma virus (HPV) as a tool for cervical cancer screening for women aged 30 to 65, with an interval of five years or more	☐	☐	☐	☐	☐
Quantitative faecal immunochemical testing (FIT) as a screening test for colorectal cancer before referring individuals between 50 and 74 years old to follow-up colonoscopy	☐	☐	☐	☐	☐
Low-dose computed tomography as a screening test for individuals at high risk for lung cancer, including heavy smokers and ex-smokers	☐	☐	☐	☐	☐
Prostate cancer screening for men, on the basis of prostate-specific antigen (PSA) testing in combination with magnetic resonance imaging (MRI) scanning as follow-up	☐	☐	☐	☐	☐
Screen-and-test strategies for Helicobacter pylori for gastric cancer.	☐	☐	☐	☐	☐

D5. How would you assess the relevance of the following preventive EU cancer plan recommendations, specifically in the field of rare cancers?

Please use a scale of 1 to 5. A score of 1 indicates that the recommendation is not relevant for rare cancers, while a score of 5 indicates strong relevance for rare cancers.

	1	2	3	4	5
Creation of a Knowledge Centre on Cancer to facilitate the coordination of scientific and technical cancer-related initiatives at EU level	☐	☐	☐	☐	☐
Enabling cancer patients to securely access and share electronic health records for prevention and treatment across borders through the European Health Data Space	☐	☐	☐	☐	☐
Elimination of cancers caused by human papillomaviruses through EU support for Member States on vaccination with the aim to vaccinate at least 90% of the EU target population of girls and to significantly increase the vaccination of boys	☐	☐	☐	☐	☐
Improvement of health literacy on cancer risk by updating the European Code against cancer	☐	☐	☐	☐	☐
Creation of a 'Tobacco-Free Generation', including reviewing the Tobacco Products and the Tobacco Taxation Directives and the legal framework on cross-border purchases of tobacco	☐	☐	☐	☐	☐
Reducing the harmful alcohol consumption through support to capacity-building and best practice; reduce young people's exposure to online marketing and advertising of alcohol products; implement evidence-based brief interventions	☐	☐	☐	☐	☐
Addressing unhealthy diets, obesity and physical inactivity by reducing carcinogenic contaminants in food	☐	☐	☐	☐	☐

	1	2	3	4	5
Alignment of the EU's air quality standards more closely with the WHO guidelines and promote sustainable and smart mobility	☐	☐	☐	☐	☐
Adoption of a new Occupational Safety and Health Strategic Framework to further reduce workers' exposure to chemicals	☐	☐	☐	☐	☐
Developing a new EU Cancer Screening Scheme to ensure that 90% of the target population is offered breast, cervical and colorectal cancer screening	☐	☐	☐	☐	☐

Section E: CLINICAL MANAGMENT, RESEARCH AND INOVATION

E1. To what extent are evidence-based treatment guidelines for rare tumors available at your center?

Use a scale of 1 to 5, with 1 indicating there are no evidence-based treatment guidelines and 5 indicating the availability of treatment guidelines for all rare cancer patients at the center.

1 ☐
2 ☐
3 ☐
4 ☐
5 ☐

E2. How would you assess the usability of the available treatment guidelines for rare cancers in your clinical unit based on the following criteria?

Please use the scale from 1 to 5, where 1 indicates strongly disagree and 5 indicates strongly agree.

	1	2	3	4	5
EFFICACY: Guidelines have demonstrated the ability to effectively treat the specific rare cancer for which they were developed.	☐	☐	☐	☐	☐
SAFETY: Guidelines have a favorable safety profile with minimal side effects, and any risks are clearly communicated to patients.	☐	☐	☐	☐	☐
FEASIBILITY: Guidelines are feasible to administer in clinical settings and do not require excessive resources or specialized equipment.	☐	☐	☐	☐	☐
PATIENT-CENTERDNESS: Guidelines take into account the unique needs and preferences of patients with rare cancers, considering quality of life and shared decision-making.	☐	☐	☐	☐	☐
NOVELTY: Guidelines offer a new or innovative approach to treating rare cancers, such as targeted therapies or personalized medicine.	☐	☐	☐	☐	☐
COST-EFFECTIVNESS: Guidelines are cost-effective, taking into account the potential benefits and harms of the treatment and the costs associated with it.	☐	☐	☐	☐	☐
EVIDENCE BASE: Guidelines are supported by a strong evidence base, including preclinical and clinical data.	☐	☐	☐	☐	☐

E3. How would you assess the <u>effectiveness of the clinical collaboration</u> in the field of rare cancers in your clinical centre based on the following criteria?

Please use the scale from 1 to 5, where 1 indicates strongly disagree and 5 indicates strongly agree.

	1	2	3	4	5
INTERDISCIPLINARY APPROACH: Collaboration involves healthcare professionals from different disciplines.	☐	☐	☐	☐	☐
COMMUNICATION: The healthcare team has open lines of communication to ensure that all team members are up-to-date on the patient's condition, treatment plan, and status.	☐	☐	☐	☐	☐
SHARED DECISION-MAKING: Collaboration involves shared decision-making between the patient, their family, and the healthcare team.	☐	☐	☐	☐	☐
EXPERTISE: Collaboration involves healthcare professionals with expertise in treating rare cancers.	☐	☐	☐	☐	☐
ACCESSIBILITY: Collaboration is accessible to patients with rare cancers, regardless of their geographic location or financial circumstances.	☐	☐	☐	☐	☐
IMPROVEMENT: Collaboration involves a continuous improvement process with regular evaluation of the effectiveness of the treatment plan.	☐	☐	☐	☐	☐

E4. How would you assess the <u>importance</u> of each of the following factors in the decision-making process for <u>reimbursement / coverage</u> of new therapies for rare cancers?

Use a scale of 1 to 5, with 1 being of minor importance and 5 being of major importance.

	1	2	3	4	5
Disease severity/clinical burden	☐	☐	☐	☐	☐
Value for money and budget impact	☐	☐	☐	☐	☐
Unmet need/lack of active treatment alternatives	☐	☐	☐	☐	☐
Strength/robustness/quality of evidence	☐	☐	☐	☐	☐
Therapeutic value (clinical efficacy/effectiveness and significance of additional benefit)	☐	☐	☐	☐	☐

E5. How would you assess the <u>effectiveness of the clinical trials for patient enrollment</u> in the field of rare cancers in your country based on the following criteria?

Please use the scale from 1 to 5, where 1 indicates strongly disagree and 5 indicates strongly agree.

	1	2	3	4	5
AVAILABILITY: There are numerous clinical trials available for different rare cancers, as well as trials for different stages of the diseases.	☐	☐	☐	☐	☐
COVERAGE: Patients have access to clinical trials regardless of where they live in the country. Trials are available in different regions, or appropriate travel and accommodation support is provided.	☐	☐	☐	☐	☐

LimeSurvey

	1	2	3	4	5
INCLUSIVITY: Clinical trials are inclusive, with patients from diverse social backgrounds and demographics being able to participate.	☐	☐	☐	☐	☐
AFFORDABILITY: Clinical trials are affordable for all patients, regardless of their financial circumstances.	☐	☐	☐	☐	☐
CONTEMPORARY: Clinical trials are characterized by a shorter duration of time between trial registration and trial initiation.	☐	☐	☐	☐	☐

E6. **How would you assess the relevance of the following clinical management EU cancer plan recommendations, specifically in the field of rare cancers?**

Please use a scale of 1 to 5. A score of 1 indicates that the recommendation is not relevant for rare cancers, while a score of 5 indicates strong relevance for rare cancers.

	1	2	3	4	5
Establishing a group of new Reference Networks on specific cancer types	☐	☐	☐	☐	☐
Creation of an EU platform to support the repurposing of existing medicines	☐	☐	☐	☐	☐
Seting up a Partnership on Personalised Medicine	☐	☐	☐	☐	☐
Developing a roadmap towards personalised prevention	☐	☐	☐	☐	☐
Launching a new project using High-Performance Computing to rapidly test existing molecules and new drug combinations	☐	☐	☐	☐	☐
Assistance of researchers working on personalised cancer treatments through tailored support and new digital platforms	☐	☐	☐	☐	☐
Supporting collaborative projects on cancer diagnostics and treatment using High- Performance Computing and AI	☐	☐	☐	☐	☐
Addressing fair access for cancer survivors to financial services (including insurance), via a code of conduct and a reflection on long-term solutions	☐	☐	☐	☐	☐
Launching a Cancer Inequalities Registry to map trends in key cancer data identifying inequalities between Member States and regions	☐	☐	☐	☐	☐
Strengthening and integration of telemedicine and remote monitoring in health and care systems; promote the virtual consultation model of the ERNs	☐	☐	☐	☐	☐
Establishing an 'EU Network of Youth Cancer Survivors	☐	☐	☐	☐	☐

E7. **How would you assess the importance of different funding sources for inovate therapy in your country for patients with rare cancers?**

This question is not seeking your subjective opinion but is about the current financing mechanisms for innovative therapy. Please rate each source on a scale of 1 to 5, with 1 indicating that the source is not used and 5 indicating that the source is a primary source of funding.

	1	2	3	4	5
Public - Goverment funding	☐	☐	☐	☐	☐
Public - Health insurance funding	☐	☐	☐	☐	☐

	1	2	3	4	5
Private - Voluntary health insurance funding	☐	☐	☐	☐	☐
Private - Household out-of- pocket expenditure	☐	☐	☐	☐	☐
Other - Non-profit institutions serving households	☐	☐	☐	☐	☐
Other - EU financing project lines	☐	☐	☐	☐	☐
Other - Industry	☐	☐	☐	☐	☐

Section F: PALLIATIVE AND SOCIAL CARE

F1. **Is palliative care <u>included in your organization's management plan</u> for patients with rare cancers?**

- Yes, provided at site ☐
- Yes, provided by other facilities ☐
- No ☐

F2. **How would you assess the <u>importance</u> of each of the following <u>medico-social support services</u> for patients with rare cancers?**

Use a scale of 1 to 5, with 1 being of minor importance and 5 being of major importance.

	1	2	3	4	5
Psychosocial support	☐	☐	☐	☐	☐
Financial support	☐	☐	☐	☐	☐
Occupational support	☐	☐	☐	☐	☐
Additional medical support (art therapy, music therapy)	☐	☐	☐	☐	☐
Spiritual and religious support	☐	☐	☐	☐	☐
Legal support	☐	☐	☐	☐	☐
Diet and nutrition support	☐	☐	☐	☐	☐

F3. **How would you assess the <u>effectiveness of palliative care</u> in the field of <u>rare cancers</u> based on the following criteria in your county?Adults rare cancer patients**

Please use the scale from 1 to 5, where 1 indicates strongly disagree and 5 indicates strongly agree.

	1	2	3	4	5
AVAILABILITY: Palliative care services are available for patients with rare cancers, including from a number of providers of hospice services and specialized palliative care teams.					
ACCESS: Palliative care services are accessible, including the ability of patients to access them regardless of their geographic location or financial circumstances.					
QUALITY OF CARE: Palliative care is provided by interdisciplinary teams based on evidence-based guidelines, and comprehensive supportive care is provided.					
TIMING: The initiation of palliative services is at the appropriate time in the disease trajectory, including early integration of palliative care in the management plan.					
PATIENT-CENTEREDNESS: Palliative care services provide personalized care plans, shared decision-making, and attention to cultural and social factors.					
SYMPTOMS MANAGEMENT: Palliative care services provide effective management of pain, nausea, fatigue, and other symptoms commonly associated with advanced cancer.					
GRIEVING SUPPORT: Palliative care services provide grief counseling and support groups.					

F4. **How would you assess the <u>effectiveness of palliative care</u> in the field of <u>rare cancers</u> based on the following criteria in your county?Pediatric rare cancer patients**

Please use the scale from 1 to 5, where 1 indicates strongly disagree and 5 indicates strongly agree.

	1	2	3	4	5
AVAILABILITY: Palliative care services are available for patients with rare cancers, including from a number of providers of hospice services and specialized palliative care teams.					
ACCESS: Palliative care services are accessible, including the ability of patients to access them regardless of their geographic location or financial circumstances.					
QUALITY OF CARE: Palliative care is provided by interdisciplinary teams based on evidence-based guidelines, and comprehensive supportive care is provided.					
TIMING: The initiation of palliative services is at the appropriate time in the disease trajectory, including early integration of palliative care in the management plan.					
PATIENT-CENTEREDNESS: Palliative care services provide personalized care plans, shared decision-making, and attention to cultural and social factors.					
SYMPTOMS MANAGEMENT: Palliative care services provide effective management of pain, nausea, fatigue, and other symptoms commonly associated with advanced cancer.					

	1	2	3	4	5
GRIEVING SUPPORT: Palliative care services provide grief counseling and support groups.	☐	☐	☐	☐	☐

F5. **How would you assess the importance of different <u>funding sources</u> <u>for social support and palliative care</u> for patient with rare cancers in your country?**

This question is <u>not asking for your subjective opinion but rather pertains to the current state of social support and palliative care</u>. Please rate each resource on a scale of 1 to 5, with 1 indicating that the source <u>is not used</u> and 5 indicating that the source <u>is a primary source</u> of funding.

	1	2	3	4	5
Public - Goverment funding	☐	☐	☐	☐	☐
Public - Health insurance funding	☐	☐	☐	☐	☐
Private - Voluntary health insurance funding	☐	☐	☐	☐	☐
Private - Household out-of- pocket expenditure	☐	☐	☐	☐	☐
Other - Non-profit institutions serving households	☐	☐	☐	☐	☐
Other - EU financing project lines	☐	☐	☐	☐	☐
Other - Industry	☐	☐	☐	☐	☐

F6. **If you have any <u>comments or suggestions</u>, please feel free to share them in the box below. We appreciate your time and effort in providing feedback.**

Thank you for taking the time to complete this survey. For additional inquiries or comments, please contact Kostadin Kostadinov, MD (kostadinr.kostadinov@mu-plovdiv.bg).
