# Supplementary material for: An Evaluation of Rare Cancer Policies in Europe: A Survey Among Healthcare Providers
Source: Cancers (Basel). 2025 Jan 7;17(2):164. doi: 10.3390/cancers17020164 (PMC11764363; doi:10.3390/cancers17020164)
Supplement: Supplementary file 1 [file cancers-17-00164-s001.zip › Supplement Table S1.pdf]

Supplement table S1. Number of respondents per country

Country	N
Italy	10
France	8
Czech Republic	6
Netherlands	6
Denmark	5
Spain	5
Bulgaria	4
Latvia	4
Portugal	4
Slovenia	4
UK	4
Croatia	3
Germany	3
Greece	3
Norway	3
Sweden	3
Austria	2
Estonia	2
Hungary	2
Lithuania	2
Slovakia	2
Belgium	1

Cyprus	1
Finland	1
Ireland	1
Malta	1
Poland	1
Switzerland	1
