# Supplementary material for: An Evaluation of Rare Cancer Policies in Europe: A Survey Among Healthcare Providers
Source: Cancers (Basel). 2025 Jan 7;17(2):164. doi: 10.3390/cancers17020164 (PMC11764363; doi:10.3390/cancers17020164)
Supplement: Supplementary file 1 [file cancers-17-00164-s001.zip › Supplement Table S2.pdf]

Supplement Table S2. Multi-criteria scoring of cancer plan effectiveness across countries.

Country	n	mean	95%CI
Austria	2	29.00	21.16, 36.84
Belgium	1	58.00	—
Bulgaria	4	14.00	11.23, 16.77
Croatia	3	48.67	37.68, 59.66
Cyprus	1	36.00	—
Czech Republic	6	51.83	47.87, 55.8
Denmark	5	48.40	41.1, 55.7
Estonia	2	41.00	27.28, 54.72
Finland	1	48.00	—
France	8	41.88	39.49, 44.26
Germany	3	41.33	39.6, 43.06
Greece	3	38.67	29.18, 48.16
Hungary	2	44.50	31.76, 57.24
Ireland	1	49.00	—
Italy	10	39.80	36.48, 43.12
Latvia	4	26.25	9.04, 43.46
Lithuania	2	46.00	44.04, 47.96
Malta	1	27.00	—
Netherlands	6	40.50	39.09, 41.91
Norway	3	48.00	46.87, 49.13
Poland	1	42.00	—
Portugal	4	40.00	39.2, 40.8
Slovakia	2	26.50	15.72, 37.28
Slovenia	4	57.00	53.51, 60.49
Spain	5	47.40	38.69, 56.11
Sweden	3	45.67	44.36, 46.97
Switzerland	1	42.00	—
UK	4	47.50	38.68, 56.32
